# BuShenKangShuai Tablet Alleviates Hepatic Steatosis via Improving Liver Adiponectin Resistance in ApoE^−/−^ Mice

**DOI:** 10.1155/2019/8986038

**Published:** 2019-02-13

**Authors:** Shu-chao Pang, Shuo Wang, Mei-ling Chen, Jun-ping Zhang, Yuan-yuan Wang, Hui-yun Jia, Li-yuan Bi, Hui Wang

**Affiliations:** ^1^First Teaching Hospital of Tianjin University of Traditional Chinese Medicine, Tianjin 300193, China; ^2^Tianjin University of Traditional Chinese Medicine, Tianjin 300193, China

## Abstract

BuShenKangShuai tablet (BSKS) is a Chinese herbal compound, which has been used to treat nonalcoholic fatty liver disease and cardiovascular diseases in clinic for over four decades. This study intends to explore whether BSKS administration can alleviates hepatic steatosis via improving liver adiponectin resistance in ApoE^−/−^ mice. ApoE^−/−^ mice were fed with western-type diet for 6 weeks and then were administrated with BSKS or atorvastatin for 6 weeks by gavage, and then blood and liver were collected for analysis. The results showed that BSKS attenuated hepatic steatosis, decreased blood lipids, and increased the serum level of adiponectin. We also found that adiponectin resistance in the liver was improved by BSKS, while the expression of TLR4 and NF-*κ*B p65 was inhibited, followed by the suppression of proinflammatory mediators of TNF-*α*. Our data provided evidence that BSKS was able to alleviate hepatic steatosis in vivo. The underlying mechanism of BSKS was focused on improving liver adiponectin resistance, thereby regulating dyslipidemia and inhibiting inflammatory signaling pathway.

## 1. Introduction

Data from clinical, experimental, and epidemiological studies indicate that nonalcoholic fatty liver disease (NAFLD), characterized by predominantly macrovesicular hepatic steatosis, is usually considered to be the hepatic manifestation of the metabolic syndrome [1].The overall prevalence of NAFLD has been significantly growing over the past few decades, mainly as a result of its close relationship with two major worldwide epidemics, obesity and diabetes mellitus [2]. In many cases, mortality in patients with NAFLD has dramatically risen, compared with the age- and gender-matched general population [3]. The reason is that NAFLD refers to a cluster of pathological spectrum risk factors, ranging from indolent hepatic steatosis, associated with an asymptomatic benign clinical process, to advanced liver diseases (nonalcoholic steatohepatitis (NASH) to fibrosis, cirrhosis, and hepatocellular carcinoma) and progressive cardiovascular diseases and/or metabolic diseases with higher cancer risks [4]. The exact pathogenesis of NAFLD and peculiarly the mechanisms leading to disease progression have not been thoroughly elucidated. Understanding NAFLD and its management is a crucial issue in current clinical practice.

Increasing evidence indicates that the pathogenesis of NAFLD is hastened by a disturbance in adipocytokines production [5]. Among them, adiponectin, as the most abundant and adipose-specific adipokine with an important anti-inflammatory, insulin-sensitizing, and antiatherogenic role, generally predicts steatosis grade and the severity of NAFLD. Furthermore, to some extent, adiponectin can attenuate liver inflammation and fibrosis [6]. Several studies have demonstrated that hypoadiponectinemia plays an important pathophysiological role in the progression of NAFLD [7]. Data from a large, multiethnic population-based cohort have also revealed that adiponectin levels are negatively related to hepatic steatosis even after correction for ethnicity, extrahepatic abdominal adiposity, and insulin sensitivity [8]. Taking these properties into account, it may be a potential way to prevent NAFLD through elevating serum level of adiponectin [9]. Current therapeutic strategies have focused on the indirect upregulation of adiponectin on the basis of pharmacotherapy or lifestyle modifications [6].

However, there are some contradictory results from studies where adiponectin was not associated with disease progression between patients with NASH and cirrhosis and those with hepatocellular carcinoma [10]. Indeed, studies on human or animal models have demonstrated that adiponectin increases in cirrhosis and hepatocellular carcinoma. The above-mentioned discrepancies among studies could be potentially attributed to the reduced liver function or compensative increased production of proinflammatory cytokines that may affect the increased adiponectin, independently of body composition and the presence of metabolic diseases [11, 12]. Overall evidence suggests the presence of a correlation between adiponectin levels and progressive hepatocellular damage [13]. Moreover, defective adiponectin activity has already been demonstrated in chronic liver diseases, but in this case the mechanism seems associated with adiponectin resistance, leading to hyperadiponectinemia, particularly in patients with severe fibrosis [14]. Thus, the expression of adiponectin in NAFLD, remains controversial. And the change of adiponectin activity in steatotic liver needs to be studied.

BuShenKangShuai tablet (BSKS) is a Chinese herbal compound, which has been used to treat NAFLD and cardiovascular diseases in clinic for over four decades. Our previous study has verified that BSKS could not only improve blood lipids metabolism, but also reduce the ratio of liver weight and body weight, and the ratio of white adipose tissue and body weight in high fat (HF) fed mice, fed with western-type diet (*P* < 0.05) [15]. What is more, it has also been found that BSKS could alleviate the clinical symptoms of patients with coronary heart disease angina pectoris and NAFLD in clinical research [16]. However, the mechanism of BSKS treating on NAFLD is still unclear. In this study, we try to answer above questions and intend to investigate the exact expression of adiponectin in HF-fed ApoE^−/−^ mice and whether BSKS can target on liver adiponectin resistance to attenuate the degree of hepatic steatosis in ApoE^−/−^ mice.

## 2. Materials and Methods

### 2.1. Animals

In this study, all of the animal experiments were carried out in accordance with institutional guidelines and under protocols approved by the Animal Ethics Committee of Tianjin University of Traditional Chinese Medicine (Tianjin, China). Eight-week-old male C57BL/6J mice and ApoE^−/−^ mice on C57BL/6J genetic background (20–22g) were purchased from Beijing Huafukang Bioscience Co., Ltd. (Beijing, China) (Certificate no. SCXK (Jing) 2014-0004). Mice were housed in cages (4/cage) at a constant environment (room temperature 21–24°C, room humidity 41–62%) under a 12 h light-dark cycle and received standard diet and water ad libitum.

### 2.2. Drugs

BuShenKangShuai tablet (Cat. No. TJZB-Z2008110052, Specification: 0.5g/tablet) was supplied by Pharmacy Department of First Teaching Hospital of Tianjin University of Traditional Chinese Medicine (Tianjin, China), whose quality control was on the basis of the medical institutions standards of Tianjin Food and Drug Administration. Atorvastatin tablet (Cat. No. H20051407, Specification: 10 mg/tablet) was produced by Pfizer Pharmaceutical Co., Ltd. (Dalian, China).

### 2.3. Experimental Design

After a 7-day acclimation period, C57BL/6J mice were fed with a normal diet and designated as control group (n=10), whereas ApoE^−/−^ mice were fed with western-type diet (21% fat and 0.15% cholesterol, Cat. No. H10141, Beijing Huafukang Bioscience Co., Ltd.) for 6 weeks and then were randomly divided into model group (n=10), BSKS group (n=10), and atorvastatin group (n=10). Mice in model group were administered 0.3 ml isopycnic sterile distilled water by gavage, and mice in BSKS group were administered 1365 mg/kg BSKS tablets by gavage, while mice in atorvastatin group were administered 3 mg/kg atorvastatin by gavage (BSKS and atorvastatin solution preparation: BSKS tablets and atorvastatin tablets were squashed by pestle and then were moved to a tube and dissolved in 0.3 ml isopycnic sterile distilled water for each mouse). All groups underwent intervention at a fixed time once a day.

Following 6 weeks of treatment, animals were anesthetized by injecting with 10% chloral hydrate intraperitoneally. Blood was sampled from mouse eyes (orbital canthus venous plexus) and then centrifuged at 3000 r/min for 10 min, and the serum was collected for subsequent detection. Liver tissues were dissected carefully from mice and placed in a physiological saline. Part of liver tissue was fixed in 10% neutral formaldehyde buffer solution, and the remainder was placed in Eppendorf tubes and immersed in liquid nitrogen to snap-freeze them.

### 2.4. Hematoxylin and Eosin Staining

Fixed liver tissues were dehydrated and embedded in paraffin. Five micrometer cross sections were prepared and stained with Hematoxylin and Eosin. The area of liver tissue was measured by two independent observers using image analysis software (Image-Pro Plus 6.0, Media Cybernetics, Inc., Rockville, MD, USA).

### 2.5. Blood Lipids Detection and Enzyme-Linked Immunosorbent Assay (ELISA)

Serum levels of low-density lipoproteins cholesterol (LDL-C) and high-density lipoproteins cholesterol (HDL-C) (Cat. No. A113-1 and A112-1, Nanjing Jiancheng Bioengineering Institute, Nanjing, China) were detected by double reagent direct method. Total cholesterol (TC) and triglyceride (TG) (Cat. No. A111-1 and A110-1 Nanjing Jiancheng Bioengineering Institute, Nanjing, China) were tested by single reagent COD-PAP method. The colorimetric analysis was performed by using a microplate reader (TECAN, Männedorf, Switzerland). All measurements were performed in accordance with the manufacturer's instructions, and each sample was measured in duplicate.

Level of adiponectin in blood was measured by using mouse-specific ELISA kits according to the manufacturer's instructions (Cat. No. SEA605Mu, Wuhan USCN Business Co., Ltd., Wuhan, China). Briefly, samples were incubated for 30 min at 37°C and then were exposed to biotin-conjugated detection antibody and streptavidin-HRP, respectively, for 60 min at 37°C. Stabilized chromogen and stop solution were added to terminate the reaction, then plates were read at 450 nm (OD values) within 2 hours using a spectrophotometer.

### 2.6. Western Blot Analysis

Liver tissue (~50 mg Sol) was homogenized (5,000 *μ*l/g tissue, 1:5 dilution) in ice-cold lysis buffer suitable for protein extraction and preserving phosphorylation states of proteins. Homogenates was centrifuged at 12,000 rpm for 20 min at 4°C in a microcentrifuge, then the supernatant was aspirated in a fresh tube, and protein content was performed using BSA as standards. Equal amounts of protein (30 *μ*g protein/lane) were solubilized in 4 ×Laemmli's buffer, boiled (95°C, 5 min), electrophoresed using 8-12% SDS-polyacrylamide gels in a Tris/HCl buffer system, and followed by electrophoretic transfer to a PVDF microporous membrane. The membranes were blocked for 1 h and then incubated overnight at 4°C with the following specific primary antibodies: anti-adiponectin antibody (1:1000, Cat. No. 2789s, Cell Signaling Technology), anti-adiponectin receptor 1 (AdipoR1) antibody (1:1000, Cat. No. ab126611, Abcam), anti-adiponectin receptor 2 (AdipoR2) antibody (1:1000, Cat. No. sc-514045, Santa Cruz), anti- Toll-like receptor 4 (TLR4) antibody (1:1000, Cat. No. 14358s, Cell Signaling Technology), anti- nuclear factor-kappa B p65 (NF-*κ*B p65) antibody (1:1000, Cat. No. 8242s, Cell Signaling Technology), and antitumor necrosis factor-*α* (TNF-*α*) antibody (1:1000, Cat. No. ab6671, Abcam). After incubation with appropriate secondary antibodies goat anti-rabbit IgG H&L (HRP) (1:1000, Cat. No. ab6721, Abcam) for 1 h, membranes were washed with phosphate-buffered saline three times. Specific bands of target proteins were detected using the enhanced chemiluminescence method (Syngene Chemigenius2; PerkinElmer, Waltham, MA) and quantified with densitometry (Gene Tools software; PerkinElmer). All bands were analyzed semiquantitatively with Image J software (National Institutes of Health, Bethesda, Maryland, USA).

### 2.7. Immunohistochemical Staining

Paraffin-embedded liver tissues fixed in 10% formalin were sectioned at 5-*μ*m thick and placed onto poly-L-lysine-coated slides. Coverslips were incubated for 1 h at 60°C and then processed by conventional dewaxing. After incubation with 0.3% H_2_O_2_ for 30 min to block endogenous peroxidase activity, the sections were washed 3 times with sterile distilled water, treated with normal goat serum for 30 min, and then incubated with primary antibodies at 4°C overnight. Immunohistochemical staining was conducted using antibodies against TLR4 (1:50, Cat. No. ab47093, Abcam), NF-*κ*B p65 (1:1000, Cat. No. ab7970, Abcam), and TNF-*α* (1:200, Cat. No. ab6671, Abcam) as primary antibodies. After being rinsed with PBS 3 times (10 min each), the slides were incubated with the secondary antibody goat anti-rabbit IgG H&L (HRP) (1:1000, Cat. No. ab6721, Abcam) for 45 min at 37°C and then washed with PBS 3 times (10 min each). The sections were incubated in the horseradish peroxidase streptavidin for 15 min at 37°C. After being washed with PBS 3 times (10 min each), the sections were stained with DAB- H_2_O_2_. The sections were counterstained lightly with hematoxylin, dehydrated in ethanol, cleared in xylene, mounted with neutral rubber sealant, and then observed under the bright field microscope (Olympus, CX21). Pictures were counted in 5 microscopic fields chosen randomly at ×400 magnification under the microscope. Image analysis software (Image-Pro Plus 6.0) was used to calculate the ratio of positive to all areas, and the average value was taken.

### 2.8. Statistical Analysis

All parameters were expressed as mean ± S.D. Statistical analysis was performed using one-way analysis of variance (ANOVA) followed by Fisher's Least Significant Difference (LSD) test for multiple comparisons. All analyses were performed using the SPSS 15.0 statistical software (SPSS Inc., Chicago, IL, USA). A value of* P* < 0.05 was regarded as statistically significant.

## 3. Results

### 3.1. BSKS Alleviates Hepatic Steatosis in ApoE^−/−^ Mice

**Figure 1:**
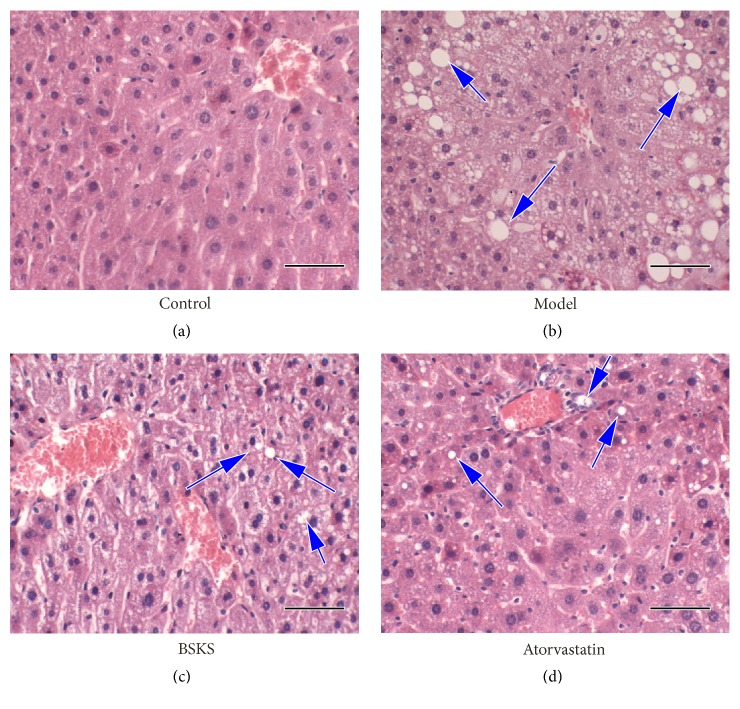
Effect of BuShenKangShuai tablet (BSKS) on the hepatic steatosis in ApoE−/− mice. Representative photomicrographs of Hematoxylin and Eosin staining of liver. The area of liver tissue was measured by Image-Pro Plus 6.0 (magnification, ×400). (a) No significant hepatic steatosis could be found in control group. (b) Hepatocyte nuclear ballooning, hepatocyte apoptosis, Mallory's hyaline, and inflammation foci were the main types of hepatic histopathological changes in model group. (c) The above histopathological changes were significantly improved in BSKS group. (d) Atorvastatin could also significantly alleviate the above histopathological changes. Except for this, the hepatic cells were arranged more closely in atorvastatin group. Scale bars 50 *μ*m.

To explore whether BSKS can improve the histopathological changes in hepatic steatosis caused by western-type diet, we examined liver tissues of mice with pathological section staining (Figure 1). The results of H&E staining have shown that the basic structure of the hepatic lobule was still complete, and no significant hepatic steatosis could be found in the control group. Compared with the control group, hepatocyte nuclear ballooning, hepatocyte apoptosis, Mallory's hyaline, and inflammation foci were the main types of hepatic histopathological changes in the model group. After the treatment (BuShenKangShuai tablet or atorvastatin), the histopathological changes were significantly improved, especially in the atorvastatin group, in which the hepatic cells were arranged more closely.

### 3.2. BSKS Decreased Blood Lipids Level

**Figure 2:**
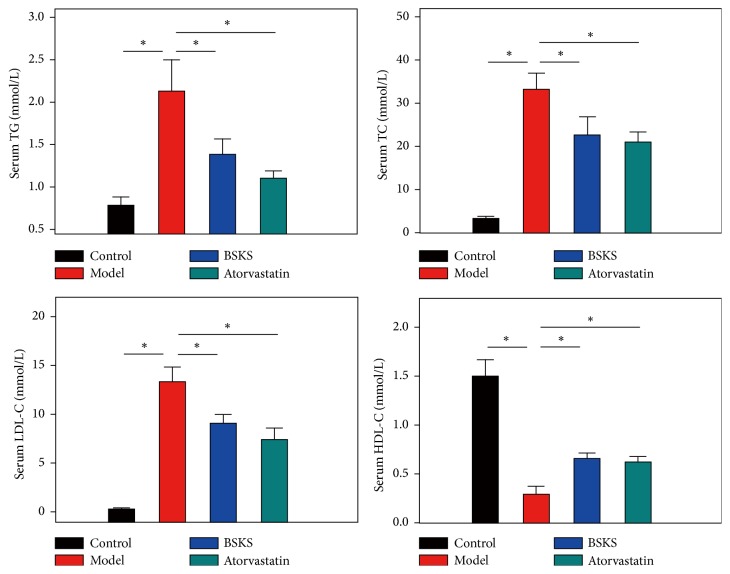
Effect of BSKS on blood lipids. The serum TG, TC, LDL-C, and HDL-C of mice were detected and compared between the control group and model group, model group and drug treatment groups (*n* = 10). Data are shown as mean ± S.D and compared by one-way analysis of variance (ANOVA) followed by Fisher's Least Significant Difference (LSD) test for individual comparisons. ^n.s.^*P* > 0.05; ^⋆^*P* < 0.05.

The imbalance in lipids metabolism is tightly associated with NAFLD, representing an emerging health concern. Lipid deposition within hepatocytes accumulation, resulting from a disparity between lipids availability and lipid disposal in the liver, is described as hepatic steatosis [17]. To further prove the antihepatic steatosis effect of BSKS, we next evaluated circulating lipids level (Figure 2). The result demonstrated that serum TG, TC, and LDL-C in the model group were significantly higher (*P* < 0.05), but the level of HDL-C in the model group was much lower (*P* < 0.05), in contrast with the control group. After drug administration treatment for 6 weeks, we found that BSKS showed similar lipid-lowering effect with atorvastatin, which significantly reduced serum TG, TC, and LDL-C and increased HDL-C (*P* < 0.05). These results provided evidence that the anti-hepatic steatosis effect of BSKS might be due to improving blood lipids metabolism.

### 3.3. BSKS Promoted the Binding Sensitivity of Adiponectin to Its Receptor

**Figure 3:**
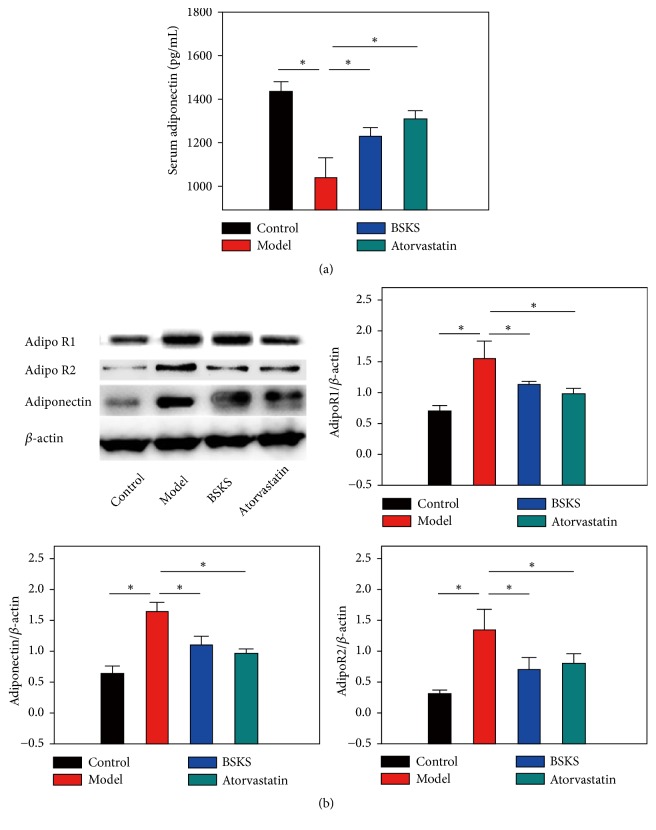
Effect of BSKS on the serum level of adiponectin and the expression of adiponectin and its receptors in the liver tissue. (a) The serum level of adiponectin was detected by ELISA (*n* = 10). (b) The expressions of adiponectin, AdipoR1, and AdipoR2 in the liver were detected by western blot (*n* = 3). The results were quantitatively compared between the control group and model group, model group and drug treatment groups. Data are shown as mean ± S.D and compared by ANOVA followed by LSD test for individual comparisons. ^n.s.^*P* > 0.05; ^⋆^*P* < 0.05.

To clarify whether the expression of adiponectin in blood and liver tissues of ApoE^−/−^ mice, fed with western-type diet, was different and how it happened, we firstly performed ELISA to examine the serum level of adiponectin. Furthermore, given that adiponectin sensitivity, mediated by adiponectin/adiponectin receptors signaling, plays a significant role in the pathogenesis of NAFLD [18], we performed western blot analysis of adiponectin, AdipoR1, and AdipoR2 in adiponectin target liver tissue. In comparison to the control group, the model group expressed the lower serum level of adiponectin (*P* < 0.05) (Figure 3(a)), but higher liver tissue levels of adiponectin, AdipoR1, and AdipoR2 (*P* < 0.05) (Figure 3(b)). After treatment with BSKS and atorvastatin for 6 weeks, the serum level of adiponectin increased (*P *< 0.05) (Figure 3(a)), while the expression of adiponectin, AdipoR1, and AdipoR2 in the liver significantly decreased (*P *< 0.05), compared with the model group (Figure 3(b)).

### 3.4. BSKS Decreased Inflammatory Mediators Pathway

**Figure 4:**
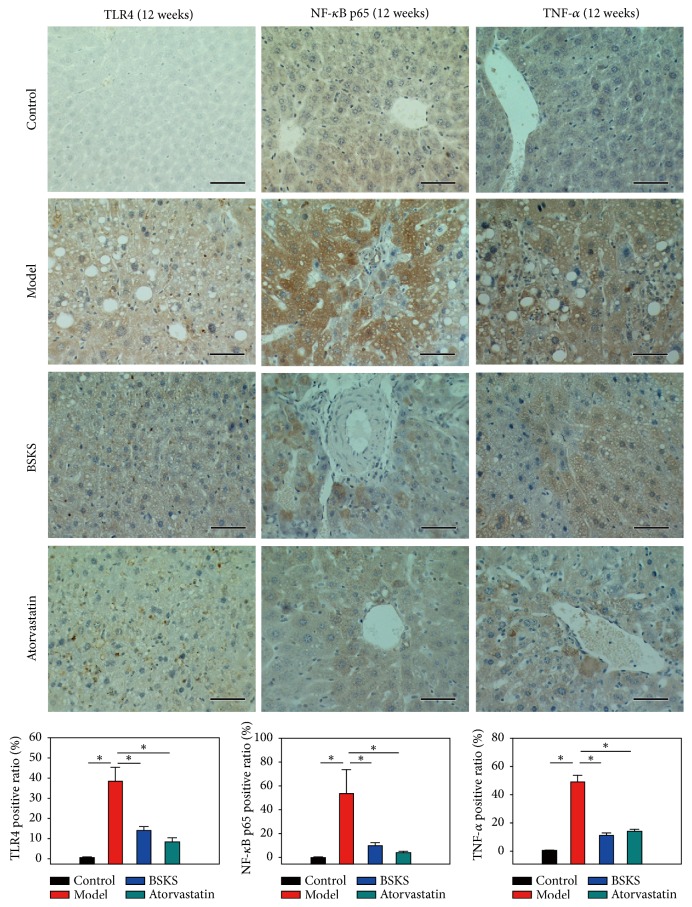
Effect of BSKS on TLR4 mediated signaling pathway, including TLR4, NF-*κ*B p65, and TNF-*α*, which were detected by immunohistochemical staining. The percentage of immunohistochemical staining area was quantitatively analyzed by using Image-Pro Plus 6.0 (magnification, ×400). The results were quantitatively compared between the control group and model group, model group and drug treatment groups (*n* = 10). TLR4 mediated signaling pathway was activated in model group. BSKS and atorvastatin could inhibit TLR4 mediated signaling pathway. Data are shown as mean ± S.D and compared by ANOVA followed by LSD test for individual comparisons. ^n.s.^*P* > 0.05; ^⋆^*P* < 0.05. Scale bars 50 *μ*m.

Inflammation is thought to be the driving force behind NAFLD and the progression to NASH, hepatic fibrosis, and subsequent cirrhosis [19]. Based on this, we tried to find whether BSKS could affect inflammatory mediator pathway. Primarily, we performed immunohistochemical staining of TLR4/NF-*κ*B p65 pathways in the liver. As demonstrated in Figure 4, the immunohistochemical staining results of TLR4, NF-*κ*B p65, and TNF-*α* in the liver showed that, in the control group, there were a small number of tiny tan particles scattered throughout the cytoplasm and/or the nucleus, while, in model group, the brown area was expanded, and the level of TLR4, NF-*κ*B p65, and TNF-*α* had significantly increased (*P* < 0.05). As expected, after the treatment with BSKS or atorvastatin for 6 weeks, the positive staining area of TLR4, NF-*κ*B p65, and TNF-*α* in the liver was decreased, in contrast with the model group (*P* < 0.05).

**Figure 5:**
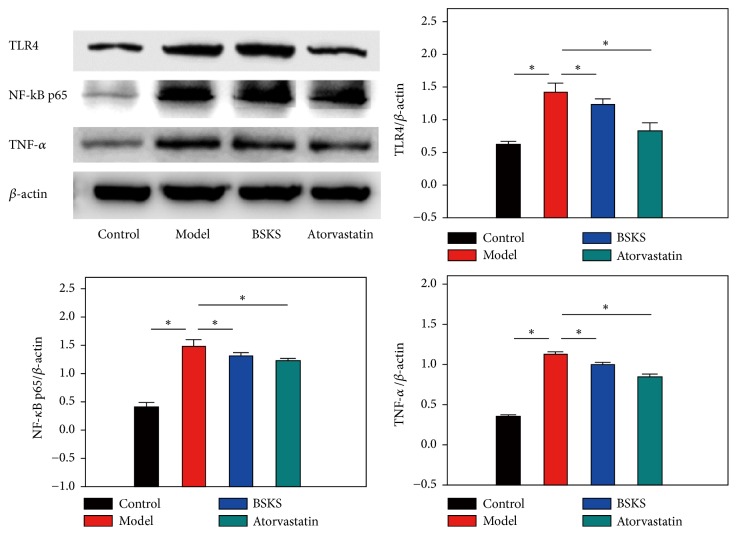
Effect of BSKS on TLR4 mediated signaling pathway, including TLR4, NF-*κ*B p65, and TNF-*α*. The results were quantitatively compared between the control group and model group, model group and drug treatment groups (*n* = 3). The western blot results were coincidentally consistent with the immunohistochemical staining results. Data are shown as mean ± S.D and compared by ANOVA followed by LSD test for individual comparisons. ^n.s.^*P* > 0.05; ^⋆^*P* < 0.05.

Furthermore, western blotting was performed to quantify the protein level of TLR4, NF-*κ*B p65, and TNF-*α* in the liver. Coincidentally, the results were consistent with the immunohistochemical staining results. We found that protein levels of TLR4, NF-*κ*B p65, and TNF-*α* were significantly increased in the model group, as compared to the control group (*P* < 0.05). Similarly, BSKS or atorvastatin treatment remarkably inhibited the protein expression of TLR4, NF-*κ*B p65, and TNF-*α*, compared with the model group (*P* < 0.05) (Figure 5).

## 4. Discussion

Obesity and especially visceral fat accumulation cause dyslipidemias and insulin resistance (IR), which are common risk factors for hepatic steatosis. Currently, the theory of systemic lipotoxicity (deleterious effects of lipid accumulation in nonadipose tissues are known as lipotoxicity [20]) has been applied to NAFLD, where excessive or dysfunctional regulation of free fatty acids (FFAs) and/or their metabolites induces hepatocytes injury and lipoapoptosis [19, 21]. The primary factor in the mechanism of the induction of dyslipidemias and IR by obesity is the abnormal expression of adipocytokines, physiologically active substances which are released by adipose tissue.

Adiponectin, an adipocytokine in inverse proportion to the body mass index that enhances the burning of fatty acids and the anti-inflammatory effect and improves IR and the pathological condition of NAFLD, can be used as an effective therapy against dyslipidemias and IR that accompanies obesity or NAFLD [18]. Adiponectin works, depending on binding to adiponectin receptors. Two seven-transmembrane domains proteins, AdipoR1 and AdipoR2, which are ubiquitously expressed, have been identified to function as its receptors and mediate increased fatty acid oxidation and glucose uptake by adiponectin. AdipoR1 is abundantly expressed in heart and skeletal muscle, whereas AdipoR2 is supposed to be the main receptor in the liver, suggesting an association with the pathology of liver diseases [22]. Some studies [18, 23] have shown that hepatic adiponectin sensitivity and resistance mediated by adiponectin/AdipoR signaling play a vital role in the pathogenesis of NAFLD. Evidence of adiponectin resistance has also been shown in obesity and following chronic HF-fed conditions. Customarily, people believe that the decreased expression of AdipoR1 and/or AdipoR2 leads to a decrease in adiponectin binding, and this in turn leads to decrease effects of adiponectin, termed adiponectin resistance, the so-called vicious cycle [24]. Based on this, elevating the expression of AdipoR1 and/or AdipoR2 should be a useful treatment to improve adiponectin resistance.

However, this study showed that the model group, fed with western-type diet for 12 weeks, expressed lower serum level of adiponectin (*P* < 0.05), but higher liver tissue protein levels of adiponectin, AdipoR1, and AdipoR2 (*P* < 0.05), in contrast with the control group. While the serum level of adiponectin was upregulated, the expression of adiponectin, AdipoR1, and AdipoR2 in the liver was decreased by BSKS treatment. Changes in adiponectin and its receptors protein content do not appear to be a likely cause of adiponectin resistance. The elevated adiponectin level with adiponectin resistance is a compensatory response under the condition of an unusual discordance between IR and adiponectin unresponsiveness. Therefore, we cannot rule out the possibility that adiponectin, AdipoR1, and AdipoR2 conformation or association with the plasma membrane or other required molecules could have been altered by the HF-diet. These indicate that the low ability of adiponectin and/or low sensitivity of adiponectin binding to adiponectin receptors may provide a novel possible molecular mechanism to induce adiponectin resistance, rather than the previously well-known recognized downregulation of adiponectin receptors to induce adiponectin resistance. The decreased adiponectin response in HF-fed animals was not always attributable to a decrease in receptor content. Similar results have already been reported in muscle of HF-fed rats [24] and adipose tissues of HF-fed mice [25].

Bobbert et al. [26] believe that diet-induced hyperlipidemia was the main cause of adiponectin resistance, and vice versa. In this study, we found that hepatic adiponectin resistance was linearly associated with hyperlipidemia. BSKS could improve liver adiponectin resistance, as well as declining blood lipids in the HF-fed mice model. Otherwise, the result of this study indicated that decreased adiponectin signaling or disruption of adiponectin receptors activity served as an upstream pathway of increased inflammation in the liver. Previous study [19] has already suggested that the presence of inflammation on liver biopsy is associated with the development of advanced fibrosis in NAFLD. The presence of acinar and portal inflammation is increasingly recognized as an important histological feature of NASH. Thus, the therapy of anti-inflammation is a useful way to improve hepatic steatosis. There is an evidence that adiponectin attenuates liver inflammation by reducing the release of proinflammatory cytokines, the activation of hepatic stellate cells, and cell death of hepatocytes [21]. The underlying mechanism of adiponectin against hepatic inflammation is due to inhibition of TLR4 mediated signaling in the liver [27]. Here, in our study, adiponectin in the blood was upregulated, and adiponectin resistance in the liver was improved by BSKS treatment, while the expression of TLR4 and NF-*κ*B p65 in the liver was inhibited by BSKS, followed by the suppression of proinflammatory mediators of TNF-*α*.

These results suggested that BSKS could alleviate hepatic steatosis in HF-fed mice, through improving the degree of liver adiponectin resistance, which might be involved in regulating dyslipidemia and inhibiting the inflammatory response via TLR4 and NF-*κ*B p65 signaling pathway.

## 5. Conclusions

This study indicated that the development of hepatic steatosis was able to be alleviated by BSKS in vivo. The underlying mechanism of BSKS was focused on improving liver adiponectin resistance, thereby improving lipid metabolism and inhibiting inflammatory signaling pathway. However, further studies are needed to illustrate which active ingredient of BSKS plays the major role in improving adiponectin resistance.

## Data Availability

The data used to support the findings of this study are available from the corresponding author upon request.
